# Understanding Solid-Based Platelet-Rich Fibrin Matrices in Oral and Maxillofacial Surgery: An Integrative Review of the Critical Protocol Factors and Their Influence on the Final Product

**DOI:** 10.3390/medicina59111903

**Published:** 2023-10-27

**Authors:** Ángel-Orión Salgado-Peralvo, Naresh Kewalramani, Alba Pérez-Jardón, Jesús Pato-Mourelo, Adriana Castro-Calderón, Lorenzo Arriba-Fuente, Mario Pérez-Sayáns

**Affiliations:** 1 Department of Dental Clinical Specialties, Faculty of Dentistry, Complutense University of Madrid, 28040 Madrid, Spain; adcast05@ucm.es (A.C.-C.); larribaf@ucm.es (L.A.-F.); 2 Department of Nursery and Stomatology, Rey Juan Carlos University, 28922 Madrid, Spain; k93.naresh@gmail.com; 3 Oral Medicine, Oral Surgery and Implantology Unit (MedOralRes), Faculty of Medicine and Dentistry, University of Santiago de Compostela, 15782 Santiago de Compostela, Spain; perlopjm@gmail.com (A.P.-J.); jpatomourelo@hotmail.com (J.P.-M.)

**Keywords:** platelet-rich fibrin, blood buffy coat, centrifugation, phlebotomy, blood specimen collection

## Abstract

Platelet-rich fibrin (PRF) is a second-generation platelet concentrate whose use in clinical practice has been widely disseminated. This has led to the development of several commercial protocols, creating great confusion as to the terminology and implications of each of them. This integrative review aims to identify the critical factors of each of the phases of the solid-based PRF matrix protocol and their possible influence on their macro- and microscopic characteristics. An electronic search of the MEDLINE database (via PubMed), Web of Science, Scopus, LILACS, and OpenGrey was carried out. The search was temporarily restricted from 2001 to 2022. After searching, 43 studies were included that met the established criteria. There were numerous factors to consider in the PRF protocol, such as the material of the blood collection tubes, the duration of phlebotomy, the parameters related to blood centrifugation, the time from centrifugation to dehydration of the fibrin clots and their dehydration into membranes, as well as the time to clinical use. These factors influenced the macro- and microscopic characteristics of the PRF and its physical properties, so knowledge of these factors allows for the production of optimised PRF by combining the protocols and materials.

## 1. Introduction

Platelet-rich fibrin (PRF) is a second-generation platelet concentrate (PC) discovered by Choukroun et al. [1] in 2001. Unlike other previously discovered PCs, such as platelet-rich plasma (PRP) or platelet rich growth factors (PRGF), PRF does not require the addition of platelet activators to the blood, such as bovine thrombin or calcium chloride [2]. This favours slow and natural fibrin polymerisation due to the physiological blood thrombin concentrations that facilitate the trapping of circulating (intrinsic) cytokines in the fibrin mesh and their gradual release. This creates equilateral junctions and a flexible fibrin mesh [3,4].

At the molecular level, PRF constitutes a fibrin mesh or network to which primarily platelets and leukocytes, and to a lesser extent circulating cytokines and stem cells adhere [5]. Platelets constitute the major component of PRF, which also contains several platelet-derived protein molecules that influence the signalling cascade in wound healing. These molecules are stored in three types of granules (alpha [α], delta, and lambda) present within platelets. The alpha granules are the most abundant, are released upon platelet activation, and contain the largest pools of growth factors (GFs) that are responsible for regenerative processes in both hard and soft tissues [6]. The most important GFs present in PRF are transforming GF-beta (TGF-ß), platelet-derived GF (PDGF), insulin-like GF-1 (IGF1), vascular endothelial GF (VEGF), and epidermal GF (EGF). In addition, PRF contains cytokines, such as interleukins (IL)-1ß, IL-6, IL-4, and tumour necrosis factor (TNF)-α, related to the modulation of inflammation and autoimmunity [7].

Due to the wide commercial diffusion of PRF and the numerous scientific investigations published in the last decade, its protocol has undergone several modifications [8]. A recent systematic review revealed the publication of 29 different protocols, with large variations in their centrifugation parameters, the use of centrifuges with different characteristics, as well as the use of blood collection tubes of different materials and volumes. This has created a great deal of confusion and has obtained heterogeneous PRF clots at the macro- and microscopic levels [9]. Among the best-known commercial protocols are L-PRF (leukocyte- and platelet-rich fibrin), A-PRF (advanced PRF) [10], A-PRF plus (A-PRF+) [11], titanium PRF (T-PRF) [12], and concentrated growth factors (CGFs). Primarily, these protocols are based on reducing the relative centrifugal force (RCF) and centrifugation time to prevent tissue loss and to achieve an optimisation of the obtained PRF clots. These types of PRF are obtained in the form of gels; however, they can also be obtained in liquid form, which was named injectable PRF (i-PRF) [13]. i-PRF can be injected or mixed with chopped PRF membranes and particulate bone graft, forming a conglomerate through the polymerisation of fibrinogen called “sticky bone” [14].

Currently, the indications of PRF in dentistry and oral and maxillofacial surgery are numerous, and all of them are related to the enhancement and acceleration of hard and soft tissue healing. These include the treatment of buccosinusal communications, osteonecrosis of the jaws, or improving the osseointegration of dental implants. PRF is widely used in sinus lift surgeries, bone regeneration, as well as in the treatment of furcation defects and intraosseous lesions, among others [15].

PRF is considered a living biomaterial [16,17], and modifications to the protocol can result in different types of PRF with different characteristics. A precise understanding of each part of the protocol is important to achieve an optimal PRF that supports the regenerative process. Therefore, the present study aims to identify the critical factors in each step of the protocol for obtaining solid-based PRF matrices and their possible influence on the characteristics of the final product.

## 2. Materials and Methods

This article is an integrative review, a kind of literature review that looks more broadly at a phenomenon of interest and allows for diverse research, which may contain theoretical and methodological literature to address the aim of the review [18].

**Figure 1:**
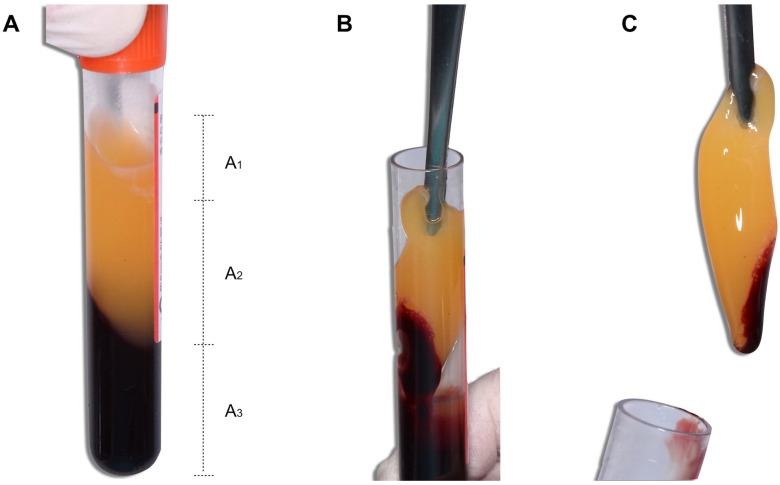
Blood collection tube after centrifugation. (**A**) After centrifugation, the blood was separated into three fractions: A1., platelet-poor plasma (PPP), A2., PRF fibrin clot, and A3., RBCs (Red blood cells) (**B**) Using surgical tweezers, the (**C**) fibrin clot was removed.

After centrifugation, the blood was separated into three factions, namely platelet-poor plasma (PPP), a PRF fibrin clot, and Red blood cells (RBCs), (Figure 1A). Using surgical tweezers (Figure 1B), the fibrin clot was removed (Figure 1C).

**Figure 2:**
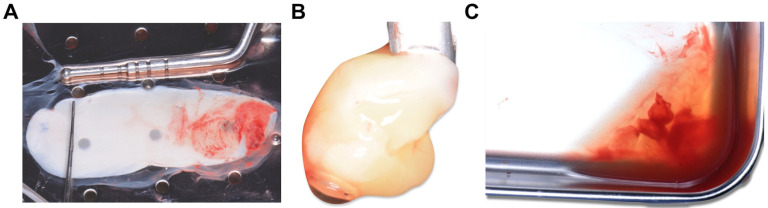
PRF derivatives. Once the PRF clot was obtained, (**A**) it was dehydrated between two flat surfaces to obtain membranes or (**B**) in a cylinder on which a piston was placed to obtain plugs. (**C**) This dehydration released an exudate rich in GFs.

Finally, the fibrin clot was dehydrated between two flat surfaces to obtain membranes (Figure 2A) or in a cylinder with a piston on top to obtain plugs (Figure 2B). The dehydration of the fibrin clots released a GF-rich exudate that could be collected with a syringe and used (Figure 2C).

**Figure 3:**
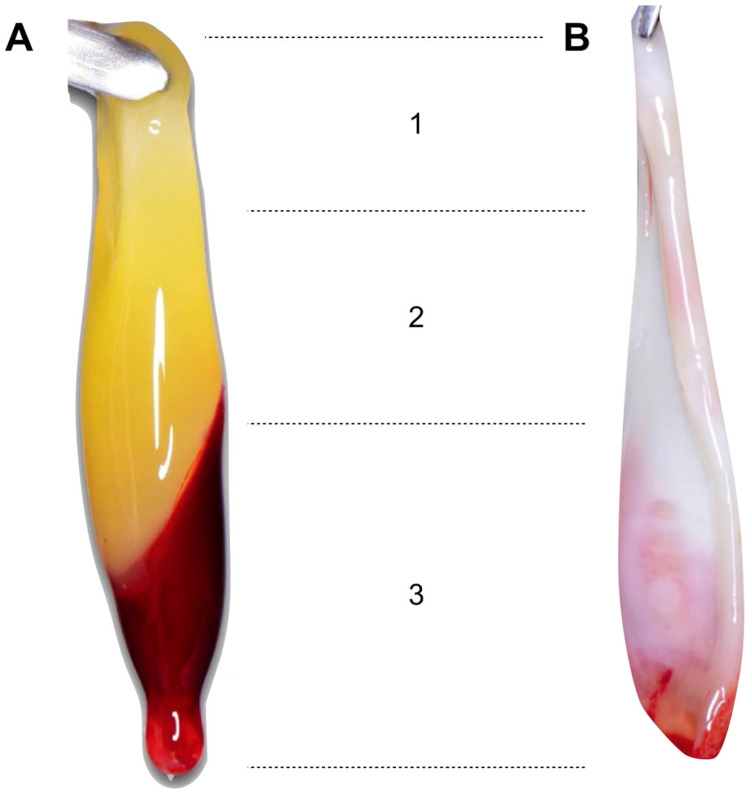
Correspondence of the (**1**) tail, (**2**) body, and (**3**) face in (**A**) a fibrin clot and in (**B**) a PRF membrane.

**Figure 4:**
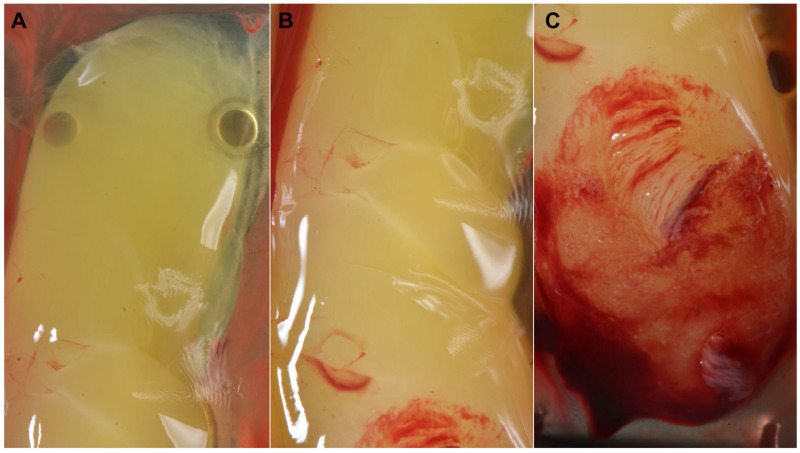
The (**A**) tail, (**B**) body, and (**C**) face in a PRF fibrin clot.

The PRF membranes were composed of three zones: the “face” (or red zone), representing the area that was in contact with the RBCs after centrifugation, the “tail” located in the upper zone, and an intermediate zone called the “body” [19,20] (Figure 3 and Figure 4).

The face contains the highest concentration of platelets and leukocytes, and the tail contains the lowest due to differences in the relative density of the blood components. White blood cells (WBCs) are the least dense compared to RBCs (1055–1085 kg/m^3^ vs. 1095–1100 kg/m^3^), while platelets have an intermediate density (1040–1065 kg/m^3^) [20].

### 2.1. Problem Formulation

Given the large number of commercial protocols that have been described in recent years, this article aims to answer the following question: “How do the different materials and factors involved in the process of obtaining PRF influence the final product at the macro- and microscopic level?*”* To this end, the phases of the PRF protocol were identified, structuring this integrative review into six steps: (1) venipuncture, (2) influence of the time from venipuncture to blood centrifugation, (3) blood centrifugation, (4) time from centrifugation to the dehydration of fibrin clots, (5) processing of the fibrin clots, and (6) time from the production of the PRF membranes to their use.

### 2.2. Literature Search

A comprehensive search of the literature was conducted in the MEDLINE (via PubMed), Web of Science, Scopus, LILACS, and OpenGrey databases. The search was performed by two researchers (Á.-O.S.-P. and M.P.-S). Medical Subject Headings (MeSH) terms, keywords, and other free terms were used with the Boolean operators (OR, NOT, AND) to combine the searches, which are shown as follows. ((platelet-rich fibrin[MeSH Terms] OR PRF[Title/Abstract] OR leukocyte- and platelet-rich fibrin[Title/Abstract] OR L-PRF[Title/Abstract] OR advanced platelet-rich fibrin[Title/Abstract] OR A-PRF[Title/Abstract] OR concentrated growth factors[Title/Abstract] OR CGF[Title/Abstract] OR advanced platelet-rich fibrin plus[Title/Abstract] OR A-PRF+[Title/Abstract] OR concentrated platelet-rich fibrin[Title/Abstract] OR C-PRF[Title/Abstract] OR titanium platelet-rich fibrin[Title/Abstract] OR T-PRF[Title/Abstract] OR fibrin mesh) NOT (injectable PRF[Title/Abstract] OR i-PRF[Title/Abstract])) AND ((centrifugation[MeSH Terms] OR centrifuge) OR blood collection tubes OR (growth factor level OR growth factors) OR thrombocyte concentrate OR leukocytes[MeSH Terms] OR PRF box OR blood platelets[MeSH Terms] OR microscopy, electron, scanning[MeSH Terms] OR low-speed centrifugation concept[Title/Abstract] OR relative centrifugal force OR cell viability OR fibroblasts[MeSH Terms] OR (fibrin[MeSH Terms] OR fibrin structure) OR membranes OR fibrinogen[MeSH Terms] OR resting time OR compression time OR (mechanical properties OR mechanical tests[MeSH Terms] OR stress mechanical[MeSH Terms]) OR (phlebotomy[MeSH Terms] OR venipuncture) OR blood collection time). The same keywords were used for all the search platforms following the syntactic rules for each database.

### 2.3. Eligibility Criteria

#### 2.3.1. Inclusion Criteria

Included studies were (1) studies conducted in humans, (2) experimental laboratory studies, (3) animal studies, (4) clinical trials, (5) multicenter studies, (6) observational studies, (7) comparative studies, (8) commentaries, (9) letters to the editor, (10) systematic reviews and meta-analyses, (11) books or chapters of books, (12) narrative literature reviews, and (13) articles published in English. The search was temporarily restricted from January 2001—as the first article on PRF was published in this year [1]—to December 2022.

#### 2.3.2. Exclusion Criteria

The following studies were excluded: (1) duplicate articles, (2) case reports, and (3) articles that did not deal with the influence of each of the described phases on the macro- and microscopic characteristics of PRF.

### 2.4. Study Records

Two researchers (Á.-O.S.-P. and M.P.-S.) compared the results to ensure completeness and removed duplicates. Then, the full titles and abstracts of the remaining papers were screened individually. Finally, the full-text articles to be included in this integrative review were selected according to the criteria described above. Disagreements over eligible studies to be included were discussed with a third reviewer (N.K.) and a consensus was reached. The reference lists of the included studies were also reviewed for possible inclusion.

## 3. Results

### 3.1. Study Selection

**Figure 5:**
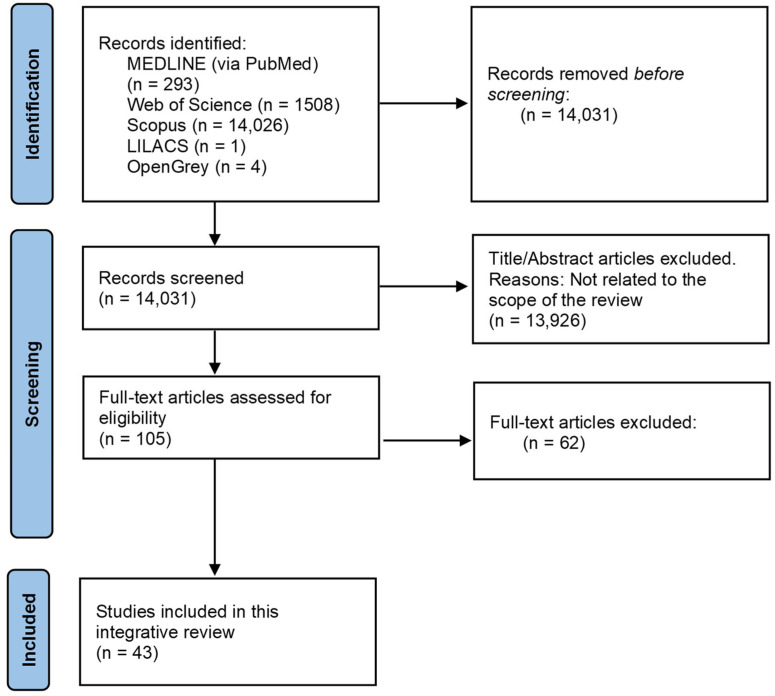
Flow diagram of the selection process.

The search strategy resulted in 15,832 results, of which 14,031 remained after removing the duplicates. Then, two researchers (Á.O.S.-P. and M.P.-S.) reviewed all the titles and abstracts and excluded 13,926 that were outside the scope of this review. Thus, we obtained 105 potential references. After reading the full text of those 105 papers, 62 were finally discarded for not dealing with some of the factors that needed to be analysed. Therefore, 43 papers were included in our integrative review (Figure 5).

### 3.2. Characteristics of the Included Studies and Pooled Results

For data processing, the results were grouped into each of the phases of the PRF protocol (Table 1).

#### 3.2.1. Venipuncture

Thirteen publications analysing the influence of blood collection tube material on the final product at the macro- and microscopic levels were included, of which nine were comparative in vitro studies conducted in humans [12,16,21,22,23,24,25,27,31], one was a letter to the editor [26], and three were literature reviews [28,29,30]. These tubes should not contain additives or platelet activators and can be plain or coated glass tubes (GTs) or plastic tubes (PTs).

##### Influence of the Blood Collection Tube Material on the Macroscopic Level

One study compared PRF clots produced with silica-coated PTs (scPTs), plain GTs, and silica-coated GTs (scGTs) after using the same fixed-angle centrifuge (RCF_max_ = 700 g; 12 min) and observed significantly larger sizes when using GTs; namely 250% larger than those produced with scPTs. Within these, the best results were obtained with plain GTs compared to scGTs (*p* < 0.05) [21]. Castro et al. [22] also observed larger sizes with GTs (1.90 ± 0.40 g) compared to scPTs (mean 1.60 ± 0.30 g), although without significant differences (*p* > 0.05), adapting the RCF to match the L-PRF, A-PRF, and A-PRF+ protocols. Other authors reached the same conclusions, observing larger clot sizes of CGFs produced in plain GTs vs. scPTs regardless of the shape of the base of the GTs (plain GTs with round base = 3.65 ± 0.49 g; plain GTs with conical base = 3.66 ± 0.37 g; and scPTs = 2.59 ± 0.38 g [*p* < 0.05]) [23].

##### Influence of the Blood Collection Tube Material at the Microscopic Level

At the morphological level, when using scPTs, the interface between the RBC layer and the fibrin clot was linear and clearly defined, whereas this interface was blurred when using GTs (plain GTs or scGTs) with RBCs being observed at the level of the face. The material also affected the distribution of platelets and leukocytes, although the results were heterogeneous. In the CGFs clots, when scPTs were used, platelets were located only at the face and leukocytes were also located in the RBC layer. In contrast, in plain GTs, platelets and leukocytes were located at the face and body level [23].

Similarly, when centrifuging scPTs using a fixed-angle centrifuge, platelets were homogeneously distributed in the CGFs clot whereas, in GTs, they were mainly located in the area distal to the centrifuge rotor. This phenomenon was explained by the fact that, in GTs, blood cells and large proteins contained in the plasma—such as the coagulation factor XII—were ejected distally by the RCF, activating the intrinsic coagulation cascade. When the end products were formed, i.e., fibrin fibres, platelets more actively aggregated with them. This coagulation also occurred in the proximal zone of the GTs but to a lesser extent. In scPTs, on the other hand, silica microparticles detached from the inner walls and were homogeneously suspended in the blood, thereby activating coagulation, and forming homogeneously distributed fibrin fibres [24].

Other authors compared the use of plain GTs and glass-coated PTs (gcPTs) in the production of PRF, concluding that the highest number of platelets and leukocytes were located in the face and, as a progression towards the body was made, they decreased until they disappeared from the first half of this area. Although the number of platelets and leukocytes were similar, they were higher when gcPTs were used (platelets: gcPTs = 7000/µL, vs. plain GTs = 6000/µL; and leukocytes: gcPTs = 3600/µL, vs. plain GTs = 3500/µL) [16].

##### Influence of Silica Coatings

Theoretically, silica microparticles released from the walls of silica-coated tubes during blood centrifugation settle to the bottom of the tube (in the RBCs layer). However, Tsujino et al. [25] found significant levels (5–30%) of silica microparticles incorporated into the fibrin matrix in three different brands of scPTs. This may have been because these particles were small enough to remain in colloidal suspension in the clot and the PPP layer [26] and may have negatively affected the regenerative process when introduced into human tissues [25]. In this regard, an in vitro study incorporated silica microparticles obtained from scPTs into cell cultures of human periosteal cells and found that they adsorbed through the cell plasma membrane with a high affinity, inducing apoptosis and reducing cell proliferation and viability. Amorphous silica is less toxic than crystalline [27]; however, both are harmful to health. During and after the preparation of the fibrin matrix, silica microparticles may also over activate or disrupt platelets and other blood cells, reducing their efficacy and therapeutic potential [28]. However, no severe complications have been reported following the application of PRF using silica products, probably due to the body’s ability to phagocytose these microparticles, or through detoxification by scavengers [29], cytoprotection by redox systems [30], or serum albumin [31].

To reduce the risk of silicosis caused by silica-coated tubes, tubes made of other materials such as grade IV titanium were developed, giving rise to T-PRF. The results showed clots with a highly organised network and a continuous integrity compared to the L-PRF samples, as well as a significantly larger size, thickness, and longer clot duration at the tissue level [12].

#### 3.2.2. Influence of the Time from Venipuncture to the Centrifugation of Blood

Four articles analysing the influence of the time from blood collection to centrifugation were included. Of these, three were comparative in vitro studies conducted in humans [21,22,32], and one was a literature review [8].

This factor played a significant role in the final product at the macro- and microscopic level, even after adapting the RCF to match the protocols (L-PRF, A-PRF, and A-PRF+). At the macroscopic level, there was an inversely proportional relationship between the elapsed time and the length of the membranes to be obtained during the first 5 min (<1 min = 3.00 ± 0.20 cm, and 1 min = 2.40 ± 0.50 cm [*p* < 0.05]; 3 min = 1.70 ± 0.10 cm, and 5 min = 0.90 ± 0.00 cm [*p* < 0.05]). After 5 min, an amorphous clot was created and compression for the membranes was not possible [22]. At the microscopic level, scanning electron microscopy [SEM] images showed that the L-PRF clots obtained from the blood collection tubes centrifuged for less than 1 min showed clusters of platelets, leukocytes, and RBCs embedded in a well-organised fibrin matrix, whereas with increasing time, cell dispersion and a denser, more disorganised fibrin matrix occurred [22]. Therefore, ideally, the blood collection tubes should be centrifuged before the first minute [22,32] to avoid the diffuse polymerisation of fibrin [8].

One study analysed the time it took to fill the blood collection tubes, concluding that scPTs and plain GTs took approx. 15 s to fill, while scGTs took 25 s (*p* < 0.05) [21]. Rather than the tube material, this finding would be related to the vacuum system employed by each commercial firm and/or the needle calibre, which was not specified by the authors.

#### 3.2.3. Blood Centrifugation

Twenty articles dealing with the influence of blood centrifugation on PRF production were included, of which 13 were comparative in vitro studies conducted in humans [10,11,20,21,22,33,37,38,39,40,41,43,45], and two in animals [36,42], a systematic review [9], a literature review [34], a book [15], a guest Editorial [35], and a web page [44].

The centrifugation of blood separates the particles that make up the blood according to their size, density, and mass [33], with several factors influencing this process. Firstly, the blood collection tubes should be placed in pairs facing each other so that the centrifugation is symmetrical [15]. If an even number cannot be collected, a water-filled tube should be available for this purpose. Other relevant factors are the RCF, the vibration of the centrifuge, the final temperature of the tubes, their angulation during centrifugation, and the duration of centrifugation.

##### Influence of the RCF

**Figure 6:**
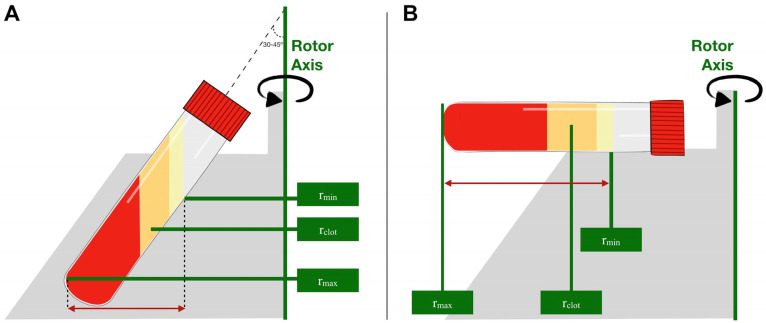
Graphic representation of centrifugation with the standard locations where the RCF is measured. (**A**) Fixed-angle centrifugation; (**B**) horizontal centrifugation.

The RCF can be defined as the amount of accelerative force applied to a blood sample in a centrifuge, and it is measured in multiples of the standard acceleration due to gravity at the Earth’s surface (g) [34]. It depends on several factors, including the speed (revolutions per minute [RPM]), rotor angulation, and rotor diameter. Regarding the diameter, the maximum radius (r_max_), i.e., the distance from the rotor to the end of the tube, and the radius from the rotor to the fibrin clot (r_clot_) must be considered. Variations in the rotor angulation, rotor diameter, or tube volume can influence the RCF. The larger the volume, the higher the r_max_ and the lower the RCF (although, in larger tubes, the cells will require a longer time to settle) [9] (Figure 6).

Therefore, knowing the RCF and r_max_ or r_clot_, we calculated the appropriate RPM, according to the desired protocol (Table 2) using the following formula. RCF = 11.18 × r × (N/1000)^2^, where “N” is the RPM and “r” is the radius measured in cm [35].

Influence of the RCF on the Macroscopic Characteristics of the PRF

Miron et al. [21] compared PRF clots produced with three centrifuges of different diameters and rotor angles (33 to 45°). The centrifuge parameters were calibrated to produce centrifugations at a low RCF (RCF_max_ = 200 g, 8 min) and a high RCF (RCF_max_ = 700 g, 12 min). The results showed that the clots obtained at a high RCF were 55–91% heavier and larger than those obtained at a low RCF. However, when comparing the clots obtained at a high RCF, those produced at the highest r_max_, and therefore at a lower RPM, produced clots that were 70% heavier and 50% larger in surface area. Other authors analysed the influence of three RCFs on PRF clots using the same time (12 min), the same centrifuge (33° rotor angulation), and scPTs and reached the same conclusions. As the RCF increased, the clot weight increased (RCF_clot_ 200 g = 5.70 g ± 0.95; 400 g = 6.40 g ± 0.95; and 600 g = 6.97 g ± 0.95) and only significant differences between 200 and 400 g were observed [36]. Thus, as the RCF decreased, the clot volume decreased [10,37].

Regarding the tensile and compressive strength, no statistically significant difference was found within the same protocol when the g-force was adapted for each device, regardless of the tube material, with values of 0.30–0.60 MPa and 0.10–0.30 MPa, respectively [22].

Influence of the RCF on the Microscopic Characteristics of the PRF

A reduced RCF resulted in a lower separation of the blood components. Despite this, homogeneous counts of platelets, lymphocytes, basophils, monocytes, and eosinophils were observed with different types of RCFs (RCF_clot_ = 200, 400 and 600 g). Decreasing the RCF (208 g, 14 min) reduced the fibrin/ RBCs count ratio to 1/1.66 [36], and the platelet counts were more homogeneous throughout the PRF clot [11,36,38]. Increasing the RCF (708 g, 12 min) increased the ratio to 1/2 [36] and platelets were sedimented and concentrated at the face level [11,36,38]. Miron et al. [20] evaluated 24 different PRF protocols (RCF_unspecified_ = 100 g, 200 g, 400 g, 700 g, 1000 g, and 1200 g, at 3, 5, 8, and 12 min) using horizontal centrifugation and observed the best distribution of platelets and leukocytes in the 3–5 mL immediately above the RBC layer at 700 g and 8 min.

Regarding the release of the GFs (VEGF, PDGF-AB and TGF-ß1) and cellular content, there were no significant differences when reducing the RCF (RCF_max_ = 653 g, 276 g, and 208 g) [22]. On the contrary, other authors observed that, at a low RCF, there was a greater release of GFs from 15 min to 10 days later [11,38]. In all the protocols used, more than 74% of platelets and more than 60% of leukocytes were found in the initial clot (*p* > 0.05) [22].

The RCF also influenced the regenerative potential of the PRF. It was observed that the PRF membranes produced at a RCF_clot_ of 200 g initially healed from the margins of the bone defect, with connective tissue infiltration observed in the central zone, whereas at 400 and 600 g, there was less connective tissue infiltration with more homogeneous bone formation. This translated into a greater regenerative potential at the bone level at a higher RCF, with significant differences at 600 g compared to 200 g (*p* = 0.032) [36], which may have been because, at a higher RCF, larger clots were produced with a denser and more polymerised fibrin mesh [21,36].

##### Centrifuge Vibration

Several authors [39,40,41] analysed the centrifuge vibration, focusing on the excitation frequency (rotational speed), since no vibration at other frequencies was noted and reached two conclusions. The first was that, as the rotational speed (RPM) increased, there was a significant increase in vibration (either with the tubes at a half or full load). In this regard, the Intra-Spin^®^ centrifuge (Intra-Lock™, Boca Raton, FL, USA) showed the least vibration. The LW-UPD8 centrifuge (LW Scientific™, Lawrenceville, GA, USA) had 4.50 times more vibration at a full load and 5.20 times more at a half load. The Salvin 1310 (Salvin Dental Specialties™, Charlotte, NC, USA) showed 6 and 6.30 times more vibration and the A-PRF centrifuge (Process™ for PRF, Nice, France) showed 6 and 6.80 times more vibration at a full and half load, respectively. However, although the clots produced were bulkier and heavier using the Intra-Spin^®^ system, no proportional relationship was found as the centrifuge vibrations increased [40,41]. Miron et al. [21] concluded similarly by noting that, after calibrating the centrifuge parameters to produce an equivalent centrifugation in various centrifuges, they obtained 15% larger clots using the Intra-Spin^®^ centrifuge compared to the Process™ for the PRF centrifuge. The second conclusion was that radial vibrations should not exceed the threshold of 1 m/s^−2^. When exceeded, there is a risk of resonance in the tubes which could cause damage to the cell contents, resulting in a poorly polymerised fibrin gel with a matrix composed of fine fibres and an amorphous cell population. Except for the Intra-Spin^®^ centrifuge (0.75 m·s^−2^), all of them exceeded this resonance threshold (LW = 2.20 m·s^−2^; A-PRF = 3 m·s^−2^; and Salvin = 4.5 m·s^−2^) [40,41]. Despite this, no reference was found in the literature to confirm these data and they should be interpreted with caution [39].

##### Temperature

As the centrifugation speed (RPM) increases, the temperature inside the tubes increases. This temperature should be between 21° and 30° C since, at lower temperatures, PRF clots will not be generated [42]. This temperature is directly proportional to the vibration of the centrifuge. In this regard, one study analysed the internal temperature of four centrifuges commonly used to obtain PRF, observing ranges that varied between 27.50° ± 0.66 to 28.83° ± 0.67, which were all within the recommended range [41].

##### Angulation of the Tubes during Centrifugation

Centrifuges with a horizontal centrifugation or with a fixed angle are currently commercially available. The latter is the most widespread [9], with the most frequent angulations being 30° to 45° [35]. As the angulation increases, the maximum radius increases significantly and, with it, the RCF [9,43]. Therefore, if horizontal centrifugation is used, it should be carried out at 2/3 the time required for centrifugation with a fixed angle [44]. If the RCF is adapted according to the angulation of the tubes, there will be no differences in the GF release, cellular content, dimensions, and mechanical properties of the obtained clots [22]. On the other hand, horizontal centrifugation produces a separation between the PRF clot and the fraction of PPP and RBCs that is perfectly delimited and horizontal while, with fixed-angle centrifugation, they present an angulation that is homologous to the angulation of the tubes. In both types of centrifuges, the highest cellular content is located in the face. In the fixed-angle centrifuges, they are also located in the portion distal to the axis of rotation. Therefore, the cellular trauma is greater, whereas, in the horizontal centrifuges, they are distributed more homogeneously along PRF [45].

##### Centrifugation Duration

The duration of centrifugation has a major impact on the separation of blood fractions [20]. If the same parameters were used (RCF_clot_= 208 g) but the centrifugation time was decreased from 14 to 8 min, the resulting PRF clot went from having a fibrin/red blood count ratio of 1/2 and a length of 3.50 cm to a ratio of 1/3 and a length of 2.50 cm, respectively. The same pattern of release for certain GFs (VEGF, TGF-ß1, and the erythroid differentiation factor [EDF]) was observed in both groups. However, the accumulated VEGF concentration at day 10 was significantly higher in the 8 min protocol compared to the 18 min protocol (773.88 ± 117.66 pg/mL, vs. 593.15 ± 114.08 pg/mL, respectively; *p* < 0.005), as well as for the EDF (1147.07 ± 164.47 pg/mL, vs. 1106 ± 57.74 pg/mL, respectively; *p* < 0.05), with no differences in the accumulated TGF-ß1 concentration at day 10 (8 min = 36.29 ± 5.73 ng/mL, vs. 14 min = 34.08 ± 3.21; *p* > 0.05). When analysing the distribution of platelets in the PRF matrices in both protocols using immunohistochemical staining with CD-61 antibodies against platelets, a similar pattern was observed with a uniform distribution of platelets in the clot [37].

#### 3.2.4. Time from Centrifugation to the Dehydration of the Fibrin Clots

Three articles were found that analysed the influence of the time from blood centrifugation to the dehydration of the fibrin clots. Two of them were comparative in vitro studies conducted in humans [22,46] and one was a letter to the editor [47].

From these articles it was concluded that the time that elapsed influenced the final size of the fibrin clot and its physical properties [22]. Ideally, they should be removed at 3–5 min as their weight and length will gradually increase and peak at this time, followed by a decrease at 7 min. The area of the PRF membranes was 28.01% and 41.33% larger after a resting period of 3 min compared to not resting or resting at 10 min, respectively [46]. Other research showed that the size differences between the PRF membranes produced during the first 60 min after centrifugation were not significant; however, they were significant when compared to those produced at 2 or 3 h (*p* < 0.05) [22]. Gradually the clot will start to “sink”, fusing with the underlying layer of RBCs, loading these cells, and weakening the mechanical properties of the PRF membranes [47]. Specifically, by comparing the membranes produced at 3 min after centrifugation with those produced at 10 min, we observed a decrease in the maximum strain (*p* = 0.049). We observed a similar decrease between 5 and 10 min (*p* = 0.031), as well as in the maximum stress between 3 min (time of maximum stress) and 7 min later (*p* = 0.042) [46].

#### 3.2.5. Processing of the Fibrin Clots

Twelve articles were found that addressed how the processing of fibrin clots influences the final PRF, of which nine were comparative in vitro studies conducted in humans [19,22,45,46,48,49,50,52,53], two were in vitro studies conducted in animal models [42,51], and one was a letter to the editor [47].

How they are dehydrated is relevant since the exudate released by the clots is rich in GFs and proteins, such as vitronectin and fibronectin [42]. Therefore, depending on the dehydration method used, a higher amount of GFs could be maintained, and there could be a lower contraction of the fibrin mesh and less damage to the platelets [47]. There are several methods for compressing fibrin clots: (1) between sterile gauze, (2) using the Kobayashi spoon, or (3) specific surgical boxes. Compressing clots with gauze absorbs GF-rich exudate, which affects the release of PDGF and TGF-ß1 [47]. The Kobayashi [48] spoon consists of compressing PRF clots between two sterile spoons joined at the handle in a way that conditions a separation between the active parts of approximately 1 mm, creating membranes of reproducible thickness and size. The lower spoon is perforated so that the GF-rich exudate can be collected and reused. The main disadvantage is that it makes the process more difficult in the case of compressing several clots, as it generates membranes one at a time. Despite its advantages over compression between gauzes, the best method is using a specific surgical box—such as the Xpression^®^ kit (Biohorizons™, Birmingham, AL, USA), PRF Box (Process™ for PRF, Nice, France) [22,42] or L-PRF Wound Box^®^, among others [42]—which allow for the simultaneous compression of a large number of clots in aseptic conditions and moist environments.

This exudate was stored on a second level of the surgical box and could be collected using a sterile syringe. Controlling the dehydration of the membranes maintained the highest number of GFs, such as PDGF-AB at 20 min and 1, 4, and 168 h (*p* < 0.01), and TGF-ß1 and VEGF during the first 4 h (*p* < 0.01)—but not after 24 h and 168 h—compared to compression between sterile gauze [47,49]. This was because uncontrolled compression, such as with gauze, could damage platelets since PDGF-AB is only released by platelets and extrinsically incorporated. Conversely, the type of method used did not negatively affect the proportion of leukocytes as they intrinsically incorporate GFs, such as TGF-ß1 and VEGF [47].

As the compression time increased, more exudate was released, gradually decreasing the thickness of the PRF membranes. During 10 s of compression, there was a loss of 60% of the exudate, with less change observed after 10 s. Therefore, it was recommended to compress the membranes for at least 90 s, as this favoured a higher density of the fibrin mesh which increases its maximum tensile stress. Specifically, a peak is reached after 2 min of compression, which translates into a maximum stress seven times higher than after 10 s of compression [46].

Once the membranes were obtained, the area with the highest regenerative potential was the face, as it contained the highest concentration of leukocytes and platelets [45,50] (15.90% and 63.50%, respectively) compared to the body (0.30 and 54.70%) and tail (0.10 and 26.50%) (*p* < 0.001), a significantly lower fibrin area (62.70%, 91.20%, and 88%, respectively) [19], and the highest amount of PDGF-BB and TGF-ß1 [51]. Miron et al. [52] observed a 3–5 times higher cellular concentration and 15 times higher platelet concentration in the “face” than in the rest of the clot, namely in the 300–500 µL above the RBCs. Therefore, it was recommended to place the face of the membranes towards the area that was to be regenerated. These findings allowed for the description of a new derivative called concentrated PRF (RCF_clot_ = 408, 12 min; plain PTs), which consisted of the collection, using a sterile syringe, of this zone (0.3–0.5 mL) to obtain an injectable PRF with a higher concentration of leukocytes (4.62- and 7.34-fold increase at 0.3 and 0.5 mL, respectively) than in the i-PRF (RCF_unspecified_ = 60 g, 3 min; 1.23-fold), as well as a significant increase in the number of platelets (1138% and 1687% vs. 200–300%, respectively) [51]. Compared to i-PRF, C-PRF induced a two- and four-fold increase, respectively, a higher cell migration, and cell proliferation at 3 and 5 days. In addition, it was associated with a two- to three-fold higher release of GFs (PDGF-AA, TGF-ß1 and EGF) [53].

#### 3.2.6. Time from the Production of the PRF Membranes to Their Use

Three articles were found that addressed the influence of the resting time of PRF membranes. Two were comparative in vitro studies conducted in humans [49,54] and one was a letter to the editor [47].

Once PRF clots were dehydrated to produce membranes, significant amounts of GFs were released during the first 20 min after compression. The in vitro studies showed a continuous release for the next 7 days [47,49,54]. At present, it is not known how long viable PRF can be maintained outside the body before clinical use. It seems plausible that if it is kept properly hydrated, it could be used for approximately 300 min after dehydration [54]. To this end, it is recommended to collect the released exudate using a sterile syringe and, from time to time (every 20–30 min), rehydrate the PRF membranes [47], thus avoiding excessive dehydration until use, which could affect the release of GFs.

## 4. Discussion

The data presented in the present article were of great clinical relevance as they helped provide a better understanding of the protocol phases to achieve further optimisation of the solid-based PRF matrices that were produced.

One of the benefits of understanding the described variables—such as a decrease in the RCF, which was termed the “low-speed centrifugation concept” (LSCC)—was an increase in the number of leukocytes and platelets. The increased number of leukocytes enhanced hard and soft tissue regeneration by contributing to the release of the angiogenic and lymphogenic factors responsible for cellular interaction in the tissue regeneration process [55,56]. They were also involved in the communication between the precursor cells and mesenchymal cells involved in bone formation and the cell–cell communication necessary for tissue regeneration. Platelets contain GFs involved in the regenerative process that were released only after aggregation [33]. On the other hand, the LSCC resulted in significantly smaller PRF clots compared to the high-RCF protocols [21]. As the centrifugation speed decreased, the relative separation of the PRF fractions decreased, producing fibrin meshes with a lower density [10,37].

Additionally, horizontal centrifugation could improve the antibacterial properties of PRF, probably due to the increased number of immune cells in the membrane. However, its activity against *Pseudomonas aeruginosa*, *Streptococcus pneumoniae*, *S. mitis, Haemophilus influenzae*, *Escherichia coli*, and *Staphylococcus aureus* is very low. Therefore, the use of PRF as a drug delivery system against these microorganisms has been studied. In this regard, a recent investigation studied the addition of topical antibiotics to blood collection tubes prior to centrifugation and observed that 1 mg/mL of gentamicin sulfate had a massive antibacterial activity for more than 4 days against all the tested microorganisms compared to the PRF control group (*p* = 0.031). The addition of 2 mg/mL of linezolid showed no added benefit (*p* = 0.218) [57]. Other authors using a similar methodology during the experimental period—4 days after preparation—showed an antibacterial effect against *Fusobacterium nucleatum* and *S. aureus* by adding 5 mg/mL of metronidazole, 150 mg/mL of clindamycin, or 1 mU/mL of penicillin to PRF [58]. It did not appear that the compression of the PRF clot to the membrane affected the release of the antimicrobials [58]. However, high concentrations may have negatively affected the physical properties of the PRF that was obtained [57,58], so further studies are needed.

Another variable for assessing in the production of PRF was blood composition. Normal counts of the major components of PRF ranged from 140,000–340,000 platelets/µL and 4500–13,500 leukocytes/mm [59]. This meant that, even within the normal range, very high counts would be close to thrombocytosis or leukocytosis, respectively, and very low amounts would be thrombocytopenia or leukopenia. Therefore, the blood composition varied from individual to individual.

The fibrinogen levels were important as they influenced the fibrin clot properties (structure, formation time, and fibrinolysis resistance) [19]. An animal model study observed, using a linear regression analysis, a significant positive effect on the PRF membrane area on the fibrinogen (Pearson correlation coefficient [r] = 0.802; *p* = 0.02), WBCs (r = 0.625; *p* = 0.03), and platelets (r = 0.839; *p* = 0.001). However, there was no apparent correlation between haemoglobin (r = 0.514; *p* = 0.087), RBCs (r = 0.548; *p* = 0.065), and haematocrit (r = 0.454; *p* = 0.138) [60]. Miron et al. [61] observed in vivo a larger PRF membrane size in women and patients older than 65 years due to higher haematocrit counts in these patients. Therefore, there were differences not only concerning age but also gender. Thus, significantly higher fibrinogen levels were observed in women (321.30 ± 60.70 mg/dL; mean age = 29 ± 1.90 years) compared to men (269 ± 30.60 mg/dL; mean age = 29.60 ± 1.80 years) (*p* < 0.05). A higher release of BMP-9 at day one (39.30 ± 15.10 pg/mL vs. 27. 60 ± 11 pg/mL, respectively; *p* < 0.05) [19] was found while, in men, a significantly higher release of cytokines (PDGF-BB, TGF-ß1 and IGF-1) was found [51]. On the other hand, there were no gender differences in the peripheral blood platelet and leukocyte concentrations [19] or in the release of other GFs [19,22]. Some studies observed a significant positive correlation between bone morphogenic proteins 9 (BMP-9) (at 3 and 7 days) and PDGF-AB (at 7 days) and the number of platelets in peripheral blood [19], as these GFs were released by platelet alpha granules [5].

In healthy individuals, as age advanced, the aptitude of blood coagulation also increased because of the increase in the plasma concentration of most of the pro-coagulation factors [62], such as the fibrinogen levels, which increased by 10 mg/dL per decade [63]. One study showed values of 312.30 ± 70.90 mg/dL in patients aged 18 to 40 years vs. 418 ± 93 mg/dL in patients aged 60 to 80 years (*p* < 0.001) [64]. Likewise, deficits in sex steroid hormones associated with post-menopause, such as 17ß-estradiol, also significantly affected the fibrinogen levels [65].

On the other hand, the patients included in the various studies had ideal systemic conditions. They were ASA 1 [14,21,23,41,43], non-smokers [21,23,32,33,40,43,49] who did not ingest alcohol [33] or certain drugs that could interfere with coagulation—such as antibiotics, anticoagulants or antiplatelet agents, among others [14,16,21,23,32,40,41,43]—from 2 weeks [16,32] to 3 months [14,22] before the studies. Therefore, the influence of other idiosyncratic conditioning factors on the patients closest to those who usually attended the consultations on the macro- and/or microscopic characteristics of the PRF was not known. Concerning this, factors such as smoking, body mass index, total and low-density lipoprotein (LDL) cholesterol, triglycerides, blood pressure, and WBC count influenced the blood fibrinogen level [66]. Ockerman et al. [67] observed in vitro that low doses of anticoagulant (enoxaparin) did not significantly affect the mechanical properties, fibrin mesh, or cellular content of PRF, whereas high doses prevented its generation. Specifically, as the anticoagulant concentration increased, the mechanical properties and the platelet and leukocyte counts decreased, although the WBC (lymphocytes, neutrophils, eosinophils, basophils, and monocytes) counts were not significantly affected. Specifically, the control group had 72% platelets and 51% leukocytes in the whole blood sample and a more regular fibrin structure, while the test group (blood supplemented with five IU enoxaparin in 0.5 mL normal saline solution) had 47% platelets and 67% leukocytes, as well as an irregular structure.

The term “biological signature” was described to refer to the quantity and duration of the slow release of GFs [68], so each commercial protocol had its own. Given the above-mentioned inter-subject variations, it was difficult to assess to what extent it was possible to refer to a “true” biological signature. For this reason, commercial companies recommend using all their products in the process of obtaining PRF. According to the data shown in the present study, an optimised PRF could be achieved by combining the products of different brands and/or protocols (e.g., the Intra-Spin^®^ centrifuge [RCF_max_ = 653 g, 12 min] with Process™ for PRF plain GTs; or Process™ for the PRF centrifuge and Process™ for PRF plain GTs at an RCF_max_ = 700 g, 12 min of the Intra-Lock™ protocol [21], among others). This optimisation of PRF could be achieved through products with appropriate features in compliance with current regulations, without subjecting the clinician to a commercial firm—which was recently termed “free protocol” [69]—thereby reducing associated costs, favouring the access of professionals to an advanced technique and, therefore, improving the quality of care offered to patients. To this end, Miron et al. [35] alluded to the importance of specifying (1) the dimensions of the rotor (radius at the clot and the end of the tube), (2) the rotor angulation for the tube holder, (3) the RPM and time, (4) the RCF value calculated at either the RCF_min_, RCF_Clot_, or RCF_max_, (5) the composition and size of the tubes used to produce PRF, and (6) the centrifugation model used in the studies. Normally, the international guidelines for reporting the RCF values during centrifugation are most commonly reported at the RCF_max_ [70].

These recommendations aim to achieve “reproducible” results and to gain a deeper understanding of the results from the combination of the protocols. In this regard, it has been observed that, if the RCF is adapted for a given protocol, regardless of the centrifuge used, there will be no in vitro differences in the GF release, cellular content, dimensions, and mechanical properties of the PRF membranes that are obtained [22]. However, it is important to consider that the cellular crosstalk and enzymatic degradation of the fibrin network would be different in vivo.

Therefore, controlled clinical studies are needed to evaluate the regenerative potential of different PRF protocols. It is also recommended that future lines of research investigate the influence of the drugs or systemic conditions affecting the coagulation process on the macro- and microscopic characteristics of PRF clots.

## 5. Conclusions

There were numerous factors to consider in the protocol for obtaining PRF, as they influenced its macro- and microscopic characteristics and physical properties.

- Larger PRF clots were obtained using plain GTs. The material of the blood collection tubes also influenced the distribution of platelets and leukocytes in the fibrin clot.
- The time from venipuncture to centrifugation should ideally be <1 min to achieve larger fibrin clots with a higher density and organisation of the fibrin mesh.
- By adapting the RCF for a given protocol, reproducible results can be obtained. Increasing the RCF increased the size of the fibrin clots with a denser and more polymerised fibrin mesh.
- Once the blood has been centrifuged, a 3–5 min rest period is recommended before removing the fibrin clots, as this has a positive effect on their size and physical properties.
- The use of specific surgical boxes is recommended for the dehydration of the fibrin clots in membranes. It is recommended to compress them for 2 min to achieve optimal dehydration and better physical properties.
- The “face” of the membranes is the area with the highest regenerative potential, so it will be placed towards the bed to be regenerated.
- Once the PRF membranes have been produced, they can be used for the next 300 min by adequately hydrating them from time to time using the GF-rich exudate.

Therefore, knowledge of these factors will allow for the production of optimised PRF by combining the protocols and materials without relying on a commercial company.

## Figures and Tables

**Table 1:** Articles included in the integrative review based on the information provided, according to the stage of the PRF protocol.

Author	Year	Type of Study	Phase of the PRF Protocol Analysed					
			1	2	3	4	5	6
Pavlovic et al. [8]	2021	Literature review		X				
Herrera-Vizcaina, C [9]	2021	Systematic review			X			
Ghanaati et al. [10]	2014	Comparative in vitro study			X			
Fujioka-Kobayashi et al. [11]	2017	Comparative in vitro study			X			
Tunali et al. [12]	2014	Comparative in vitro study	X					
Dohan Ehrenfest et al. [16]	2010	Comparative in vitro study	X					
Andrade-Aldana et al. [19]	2022	Comparative in vitro study					X	
Miron et al. [20]	2020	Comparative in vitro study			X			
Miron et al. [21]	2020	Comparative in vitro study	X	X	X			
Castro et al. [22]	2021	Comparative in vitro study	X	X	X	X	X	
Bonazza et al. [23]	2016	Comparative in vitro study	X					
Yamaguchi et al. [24]	2020	Comparative in vitro study	X					
Tsujino et al. [25]	2019	Comparative in vitro study	X					
O’Connell, SM [26]	2007	Letter to the editor	X					
Masuki et al. [27]	2020	Comparative in vitro study	X					
Komosa et al. [28]	2017	Literature review	X					
Bodega et al. [29]	2019	Literature review	X					
Ye et al. [30]	2015	Literature review	X					
Moran et al. [31]	2002	Comparative in vitro study	X					
Esfahrood et al. [32]	2020	Comparative in vitro study		X				
Choukroun et al. [33]	2018	Comparative in vitro study			X			
Salgado-Peralvo et al. [15]	2020	Book			X			
Peck et al. [34]	2016	Literature review			X			
Miron et al. [35]	2019	Guest editorial			X			
Tovar et al. [36]	2021	Animal in vitro study			X			
El Bagdadi et al. [37]	2019	Comparative in vitro study			X			
Kobayashi et al. [38]	2016	Comparative in vitro study			X			
Dohan-Ehrenfest et al. [39]	2014	Comparative in vitro study			X			
Dohan-Ehrenfest et al. [40]	2018	Comparative in vitro study			X			
Pinto et al. [41]	2014	Comparative in vitro study			X			
Crisci et al. [42]	2017	Animal in vitro study			X		X	
Miron et al. [43]	2019	Comparative in vitro study			X			
Block Scientific [44]	2019	Web page			X			
Fujioka-Kobayashi et al. [45]	2021	Comparative in vitro study			X		X	
Wei et al. [46]	2022	Comparative in vitro study				X	X	
Dohan-Ehrenfest et al. [47]	2010	Letter to the editor				X	X	X
Kobayashi et al. [48]	2012	Comparative in vitro study					X	
Dohan-Ehrenfest et al. [49]	2009	Comparative in vitro study					X	X
Thanasrisuebwong et al. [50]	2019	Comparative in vitro study					X	
Bai et al. [51]	2018	Animal in vitro study					X	
Miron et al. [52]	2020	Comparative in vitro study					X	
Fujioka-Kobayashi et al. [53]	2020	Comparative in vitro study					X	
Su et al. [54]	2009	Comparative in vitro study						X

1—venipuncture; 2—influence of the time from venipuncture to blood centrifugation; 3—blood centrifugation; 4—time from centrifugation to the dehydration of fibrin clots; 5—processing of the fibrin clots; 6—time from the production of the PRF membranes to their use.

**Table 2:** Protocols for the different types of PRF.

Acronym	Protocol (Commercial Firm)	RPM ^1^	RCF_Clot_ ^2^	RCF_max_	Duration	Material of the Blood Collection Tubes
CGF	Concentrated growth factors (Medifuge^®^, Silfradent™, Sofia, Italy)	2700	UNS ^3^	UNS	2 min	Plain PTs ^5^
		2400	UNS	UNS	4 min	
		2700	UNS	UNS	4 min	
		3000	UNS	UNS	3 min	
T-PRF	Titanium platelet-rich fibrin	2800	UNS	~400 g ^4^	12 min	Grade IV titanium tubes
L-PRF	Leukocyte- and platelet-rich fibrin (Intra-Lock™, Boca Raton, FL, USA)	2700	~408 g	~653 g	12 min	scPTs ^6^
A-PRF	Advanced platelet-rich fibrin (Process™ for PRF, Nice, France)	1500	~193 g	~276 g	14 min	Plain GTs ^7^
A-PRF+	Advanced platelet-rich fibrin plus (Process™ for PRF, Nice, France)	1300	~145 g	~208 g	8 min	Plain GTs
i-PRF	Inyectable platelet-rich fibrin (Intra-Lock™, Boca Raton, FL, USA)	700	UNS	~60 g	3 min	Plain PTs
C-PRF	Concentrated platelet-rich fibrin (Not registered)	UNS	~408 g	UNS	12 min	Plain PTs

^1^ RPM—revolutions per minute; ^2^ RCF—relative centrifugal force; ^3^ UNS—unspecified by authors; ^4^ g—unit of measurement of the RCF; ^5^ PTs—plastic tubes; ^6^ scPTs—silica-coated plastic tubes; ^7^ GTs—glass tubes.

## Data Availability

Data available upon request.

## Abbreviations

PRF—Platelet-rich fibrin; PC—Platelet concentrate; PRP—Platelet-rich plasma; PRGF—Platelet-rich in growth factors; α—Alpha; GF—Growth factor; TGF-ß—Transforming growth factor-beta; PDGF—Platelet-derived growth factor; IGF1—Insulin-like growth factor; VEGF—Vascular endothelial growth factor; EGF—Epidermal growth factor; IL—Interleukins; TNF—Tumour necrosis factor; L-PRF—Leukocyte PRF; A-PRF—Advanced PRF; A-PRF+—Advanced PRF plus; T-PRF—Titanium PRF; CGFs—Concentrated growth factors; RCF—Relative centrifugal force; i-PRF—Injectable PRF; RBCs—Red blood corpuscles; PPP—Platelet-poor plasma; WBCs—White blood cells; GTs—Glass tubes; PTs—Plastic tubes; scPTs—Silica-coated plastic tubes; scGTs—Silica-coated glass tubes; gcPTs—Glass-coated plastic tubes; SEM—Scanning electron microscopy; RPM—Revolutions per minute; r_max_—Maximum radius; r_clot_—Radius from the rotor to the fibrin clot; EDF—Erythroid differentiation factor; LSCC—Low-speed centrifugation concept; r—Pearson correlation coefficient.
